# Consistently altered expression of gene sets in postmortem brains of individuals with major psychiatric disorders

**DOI:** 10.1038/tp.2016.173

**Published:** 2016-09-13

**Authors:** M M Darby, R H Yolken, S Sabunciyan

**Affiliations:** 1 Stanley Division of Developmental Neurovirology, Department of Pediatrics, Johns Hopkins School of Medicine, Baltimore, MD, USA

## Abstract

The measurement of gene expression in postmortem brain is an important tool for understanding the pathogenesis of serious psychiatric disorders. We hypothesized that major molecular deficits associated with psychiatric disease would affect the entire brain, and such deficits may be shared across disorders. We performed RNA sequencing and quantified gene expression in the hippocampus of 100 brains in the Stanley Array Collection followed by replication in the orbitofrontal cortex of 57 brains in the Stanley Neuropathology Consortium. We then identified genes and canonical pathway gene sets with significantly altered expression in schizophrenia and bipolar disorder in the hippocampus and in schizophrenia, bipolar disorder and major depression in the orbitofrontal cortex. Although expression of individual genes varied, gene sets were significantly enriched in both of the brain regions, and many of these were consistent across diagnostic groups. Further examination of core gene sets with consistently increased or decreased expression in both of the brain regions and across target disorders revealed that ribosomal genes are overexpressed while genes involved in neuronal processes, GABAergic signaling, endocytosis and antigen processing have predominantly decreased expression in affected individuals compared to controls without a psychiatric disorder. Our results highlight pathways of central importance to psychiatric health and emphasize messenger RNA processing and protein synthesis as potential therapeutic targets for all three of the disorders.

## Introduction

Schizophrenia (SCZ), bipolar disorder (BPD) and major depression (MDD) are complex neuropsychiatric disorders with etio-pathogenic factors that likely include both genetic and environmental components. Genome-wide association studies (GWAS) have identified polymorphisms that contribute to the risk of developing SCZ, BPD or MDD.^1, 2, 3, 4, 5^ SCZ and BPD are spectrum disorders with overlapping symptoms, suggesting that there is biological overlap between them. In fact, the most informative polymorphisms are similarly predictive of SCZ, BPD and MDD with correlated effects sizes for each disorder.^1, 5^ Most of the polymorphisms that associate with psychiatric disorders are outside of protein coding regions and likely alter gene expression rather than protein structure.^1, 3^ Functional analysis of genetic variants that associate with SCZ, BPD and MDD found that pathways that regulate histone methylation and thereby gene transcription are the most strongly implicated on the basis of the shared genetics across the three disorders, followed by immune and signaling pathways.^3^ However, the downstream impact of these polymorphisms on gene expression in the brain remains unclear. Gene set expression studies have reproducibly implicated a few key functions in particular disorders, but the core processes that are common across diagnostic groups and brain regions are not well established. Most expression studies of functionally related gene sets in postmortem brains have focused on a single brain region^6, 7, 8, 9, 10, 11, 12, 13^ and/or a single disorder.^6, 7, 9, 13^

Deficits associated with psychiatric disease have been discovered in nearly every brain region that has been studied. Compared with healthy controls, the volume of various brain regions including hippocampus, amygdala, thalamus, nucleus accumbens, pallidum, lateral ventricles^14^ and the supragranular cortical layers^15^ are reported to be different in SCZ. Gray and white matter deficits in SCZ have also been reported in the superior temporal gyrus, left medial temporal lobe,^16^ frontal temporal lobe, occipital lobe^17^ and other regions.^18^ Individuals with SCZ also have higher GABA neuron density in the white matter of the orbitofrontal cortex (OFC) in SCZ,^19^ and decreased connectivity between the cerebellum and the thalamus and the frontal cortex.^20^ Functional deficits in SCZ include impaired functioning of the magnocellular visual pathway,^21^ altered contextual modulation of critical responses in the primary visual cortex.^22^ Anatomical differences in the left and right superior temporal cortex, left supramarginal/angular gyrus, left postcentral gyrus and left posterior cingulate cortex correlate with the severity of auditory hallucinations.^23^ In addition, decreased cerebellar^24^ and hippocampal volume^25^ and anatomical differences in the gray mater of the frontal temporal lobe^17^ have been reported in BPD, and reduced gray matter volume in the OFC has been reported in MDD.^26^ Accordingly, previous studies have identified differences in gene or protein expression in the lateral cerebella, frontal cortex, prefrontal cortex, OFC, dorsolateral prefrontal cortex, anterior cingulate cortex, superior temporal gyrus and hippocampus in SCZ, BPD and/or MDD.^7, 8, 9, 10, 11, 12, 13, 27, 28, 29, 30, 31, 32, 33, 34, 35^ These findings suggest that molecular deficits underlying psychiatric disease are not restricted to a particular region and likely affect the entire brain.

In particular, genes that are dysregulated as a result of genetic variants are likely to be affected throughout the brain as the variants are present in every cell. On the basis of this rationale, several large expression and epigenetic studies have been conducted in peripheral blood lymphocytes.^36, 37, 38^ A comparison of the co-expression of genes in human brain and human blood revealed co-expression modules that are preserved across tissues, supporting the notion that polymorphisms will result in similar deficits in different cell types.^39^ Furthermore, the expression levels of key genes in the preserved modules tended to be more heritable than genes in modules that were not preserved. Therefore, it seems likely that mechanisms that regulate gene expression could affect multiple brain regions in the same way. We further reasoned that although not all changes in gene expression will be consistent across brain regions, those that are consistent are more likely to represent core pathways underlying the etiology of these complex disorders. We therefore examined gene expression in the hippocampus, which is part of the limbic system involved in learning and memory, and replicated these findings in a separate cohort using samples from the OFC, which has been implicated in mood disorders.^17, 26^ Our first cohort consisted of 100 hippocampal samples from the Stanley Array Collection, which includes individuals with SCZ, BPD and controls without a psychiatric disorder. Our second cohort consisted of 57 OFC samples from the Stanley Neuropathology Consortium, which includes individuals with SCZ, BPD, MDD and controls. For each diagnostic group, we identified expression differences relative to matched controls and ranked all genes according to the strength and direction of differential expression. Using the gene set enrichment analysis (GSEA) tool,^40^ which scores sets of genes according to their distribution within a ranked list, we identified gene sets containing a disproportionate number of genes with either increased or decreased expression in each diagnostic group. We identified gene sets that replicated across brain regions in each disorder and found that many of the replicated gene sets were consistently perturbed in all three disorders. We further examined 13 universal gene sets that were enriched in the same direction in both the hippocampus and the OFC in SCZ, BPD and MDD.

## Materials and methods

### Postmortem brain samples and RNA sequencing

We performed ultra-high-depth strand-specific RNA-seq using RNA isolated from 102 hippocampal samples in the Stanley Array Collection and a replication cohort of 59 OFC samples in the Stanley Neuropathology Consortium.^41^ Libraries were prepared and sequenced as detailed in the Supplementary Information. To minimize technical batch effects, each library was run on a full sequencing lane and samples from all diagnostic groups were distributed randomly across the sequencing runs. All of the raw sequencing files and topHat alignments are available for download at www.sncid.org. Two outliers were removed from each collection on the basis of the results of principal components analyses detailed in the supplement and Supplementary Figure S1. The remaining samples are well matched for demographic factors including age, sex, race, postmortem interval (PMI), pH and the side of the brain used to extract RNA (Table 1).

### Gene expression analysis

The details are presented in the Supplementary Information. In brief, we aligned sequencing reads to the human genome (hg19) using the TopHat2 short read aligner^42^ and counted the reads aligning to exons in each gene annotated in the UCSC Genome Bioinformatics hg19 knownGene table. We then used DESeq2 (ref. 43) to normalize raw read counts and identify differences in gene expression between each diagnostic group and matched controls using age, sex, brain pH and PMI as factors in the design formula and diagnosis as the condition of interest.

### Gene set enrichment and functional network analysis

The details are presented in the Supplementary Information. For each diagnostic group, we ranked all genes according to the Wald test statistic for differential expression provided by DESeq2.^43^ Using the GseaPreranked tool in GSEA,^40^ we identified gene sets that were enriched among overexpressed or under-expressed genes with a false discovery rate (FDR) *q*<0.05. We utilized the EnrichmentMap^44^ application for Cytoscape^45, 46^ to map the enriched gene sets into functional networks.

## Results

### Differential expression in the hippocampus

We sequenced 100 hippocampal samples to produce an average of 154 000 000 paired-end 100 base reads that aligned to the human genome (hg19; Supplementary Table S1). After counting the reads aligning to each gene, we used DESeq2 to compare gene expression in each diagnostic group with matched controls. We accounted for demographic variables in the differential expression analysis using a multi-factor design formula that includes sex, age, PMI and pH as covariates and diagnosis as the contrast variable. We detected expression from 21 861 genes in the hippocampus, most of which were not differentially expressed after multiple testing correction (Supplementary Figure S2). We found 12 genes that were differentially expressed in SCZ and seven in BPD (Table 2). No genes were differentially expressed in both SCZ and BPD.

To identify concerted changes in gene expression that accompany psychiatric disorders, we used GSEA^40^ to identify canonical pathway gene sets containing a disproportionate number of genes with either increased or decreased expression. For each disorder, we ranked all the genes using the Wald test statistic from DESeq2, which correlates with the amplitude and significance of expression differences, then identified gene sets that are enriched among overexpressed or under-expressed genes. We identified 89 gene sets enriched in SCZ and 170 in BPD (Supplementary Tables S2 and S3) with FDR *q*-values below 0.05.

**Figure 1:**
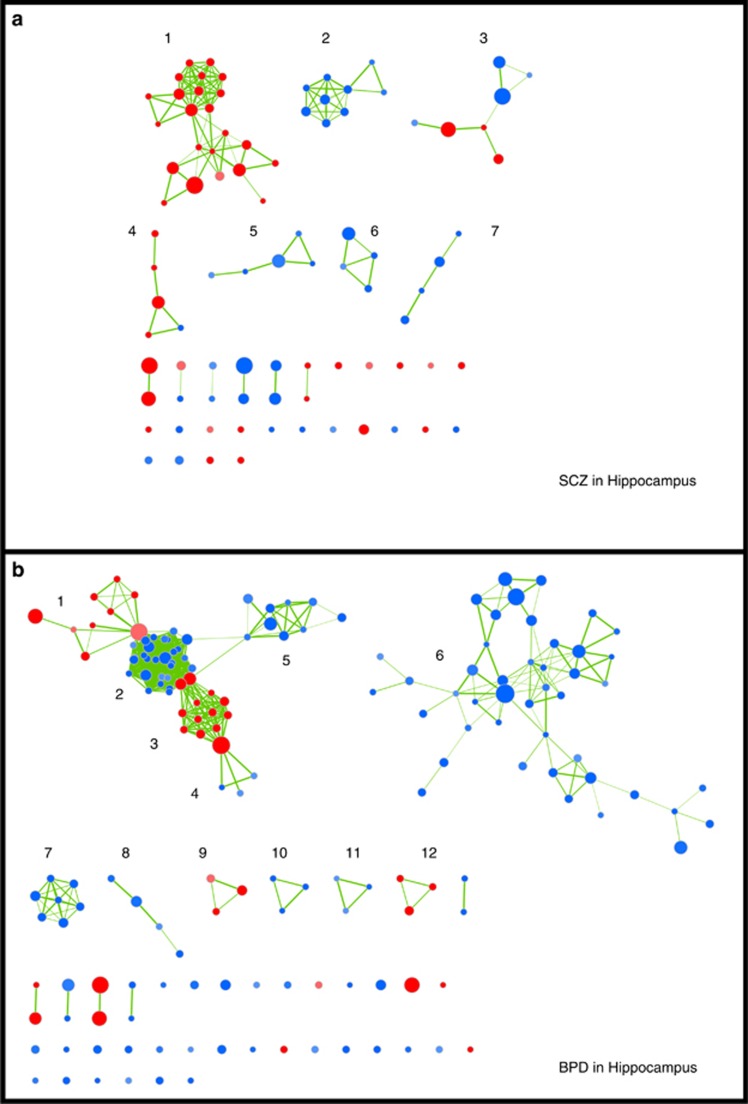
Functional analysis of overlap between sets of genes enriched in SCZ (**a**) and BPD (**b**) in the hippocampus. Red circles denote gene sets with increased expression-affected individuals, whereas blue circles indicate decreased expression. The size of each circle represents the number of genes in each set, while the thickness of the lines connecting two circles represents the proportion of genes that are in common between the two gene sets. A fully labeled version of each figure is available in the Supplementary Information (Supplementary Figures S2 and S3). The major functional networks are as follows: (**a**1) messenger RNA (mRNA) processing and translation, transport of ribonucleoproteins, transmembrane transport, interferon signaling and cytokine signaling; (**a**2) respiration/oxidative phosphorylation and genes implicated in Parkinson's, Alzheimer's and Huntington's diseases; (**a**3) developmental biology, transcriptional regulation and axon guidance; (**a**4) defensins, innate immunity and complement cascade; (**a**5) neuronal systems and neural transmitter signaling; (**a**6) extracellular matrix proteins; (**a**7) endocytosis and intracellular trafficking; (**b**1) cell cycle regulation; (**b**2) Major histocompatibility (MHC) Class I antigen presentation, ubiquitin-mediated degradation by the proteasome and related signaling networks; (**b**3) mRNA processing and translation; (**b**4) protein folding and import into mitochondria; (**b**5) transcription termination, mRNA splicing and transport, interferon and cytokine signaling; (**b**6) neuronal signaling cascades; (**b**7) respiration/oxidative phosphorylation and genes implicated in Parkinson's, Alzheimer's and Huntington's diseases; (**b**8) phospholipid metabolism; (**b**9) fatty acid and keytone metabolism, and regulation of peroxisomes and adipocites; (**b**10) transfer RNA (tRNA) biosynthesis; (**b**11) RNA polymerase III regulation; (**b**12) biological oxidations and drug metabolism by cytochrome p450. BPD, bipolar disorder; SCZ, schizophrenia.

Because we used gene sets from all major gene ontology databases, we expected many sets to be comprised of identical or highly overlapping groups of genes. We therefore utilized the EnrichmentMap application in Cytoscape^44, 45, 46^ to analyze the proportion and identity of genes from our expression data sets that are in common across the enriched gene sets (Supplementary Tables S4 and S5) and to visualize the networks created by overlapping gene sets (Figure 1, enlarged in Supplementary Figures S3 and S4). In the enrichment maps, each significant gene set from GSEA is represented by a circle. Overlapping gene sets are connected into networks by lines that represent shared genes. The size of the circles represents the number of genes in our expression data sets that are annotated in each gene set, while the thickness of the lines represents the proportion of genes that are in common between gene sets. On the basis of the number and thickness of interconnected lines, most of the gene sets that were enriched in GSEA had genes in common; they formed highly interconnected clusters describing interrelated functions. In SCZ, the largest network indicates upregulation of genes involved in messenger RNA (mRNA) processing and translation, some of which also contain genes involved in transport of ribonucleoproteins, which in turn shares genes related to other types of transmembrane transport, interferon signaling and cytokine signaling (Figure 1a, Supplementary Figure S3). In BPD, genes involved in mRNA processing and translation make up the second largest cluster. The largest cluster is of 28 highly overlapping downregulated gene sets involved in major histocompatibility (MHC) Class I antigen presentation, ubiquitin-mediated degradation by the proteasome and related signaling networks (Figure 1b, Supplementary Figure S4).

### Replication in the OFC

As we hypothesized that major molecular deficits underlying psychiatric disease would likely affect the entire brain, we performed a replication study using OFC samples from a separate cohort. We sequenced 57 OFC samples to produce an average of 140 000 000 single-end 100 base reads that aligned to the human genome (Supplementary Table S2). As with the hippocampus, we counted reads aligning to each gene and compared gene expression in each diagnostic group to matched controls using a multi-factor design that includes sex, age, PMI and pH as covariates. We detected expression from 20 711 genes in the OFC, most of which were not differentially expressed after multiple testing correction (Supplementary Figure S2). No genes were differentially expressed in SCZ, but 246 genes were significant in BPD (Supplementary Table S7) and 26 in MDD compared with controls (Supplementary Table S8).

As expected on the basis of the results of previous work,^30^ none of the individual genes that were differentially expressed in the hippocampus were replicated in the OFC. This may be, in part, because several of the genes that are differentially expressed in the hippocampus have low average read counts in the OFC (Supplementary Table S9), indicating that expression of these genes may be specific to each brain region. However, *SCL11A1, MT1X*, *S100A8* and *NR4A2* were differentially expressed in SCZ in the hippocampus and nominally significant in SCZ in the OFC. Also, *FKBP5* and *MT1X* were nominally significant in BPD in the hippocampus and significant in BPD in the OFC (Supplementary Table S9).

In contrast to individual genes, there was greater concordance between the two brain regions when expression was examined in the context of functionally related gene sets. GSEA revealed 216 gene sets that were enriched in SCZ, 491 in BPD and 257 in MDD (Supplementary Tables S10–S12) with FDR *q*-values below 0.05. As with the gene sets enriched in the hippocampus, the significant gene sets overlapped extensively to form dense interconnected networks of genes with related functions (Supplementary Tables S13–S15, Supplementary Figures S5–S7). Twenty (22.5%) gene sets enriched in SCZ and 67 (39.4%) enriched in BPD in the hippocampus were also enriched in the same direction in the same disorder in the OFC. Although some replicated gene sets were enriched only in one disorder (Supplementary Table S16), most of the gene sets that replicated across brain regions in BPD were also enriched in MDD (Table 3, Supplementary Table S17). Thirteen gene sets replicated across brain regions in both SCZ and BPD, and all of these were also enriched in MDD in the OFC (Table 3). In addition to the 13 universal gene sets, the ‘Kyoto Encyclopedia of Genes and Genomes (KEGG) Calcium Signaling' set was also replicated in both SCZ and BPD and enriched in MDD, but had decreased expression in BPD and MDD and increased expression in SCZ (Supplementary Table S16 and S17). Gene sets that are shared across diagnostic groups are less likely to be affected by medication use, which varied between diagnostic groups (Table 1). Although nearly all individuals with SCZ received antipsychotics, fewer individuals with BPD received antipsychotics and generally at lower dosages. No one in the MDD or control groups took antipsychotics. Conversely, most MDD patients used antidepressants, whereas antidepressant usage was less prevalent in the BPD and SCZ groups.

We plotted the overlap of the genes in the 13 universal gene sets (Supplementary Figure S8, Supplementary Tables S18–S22) and found that they actually denote four distinct groups. The strongest enrichment scores, particularly in BPD and MDD, were for nine gene sets involved in mRNA processing and translation (Table 3). Together, the nine overlapping gene sets contain a total of 338 genes from our expression data sets, including a core set of 80 genes that are in all the nine sets and comprise most of the ‘KEGG Ribosome' set. Expression was higher in BPD than control in 78 of the 80 genes in hippocampus and in 79 genes in the OFC. Expression was also higher in 78 of the genes in MDD. In SCZ, expression was higher in 56 genes in the hippocampus and in 52 in the OFC. The high degree of overlap (Supplementary Figure S8) suggests that a core group of genes could drive enrichment of all nine functionally related sets.

**Figure 2:**
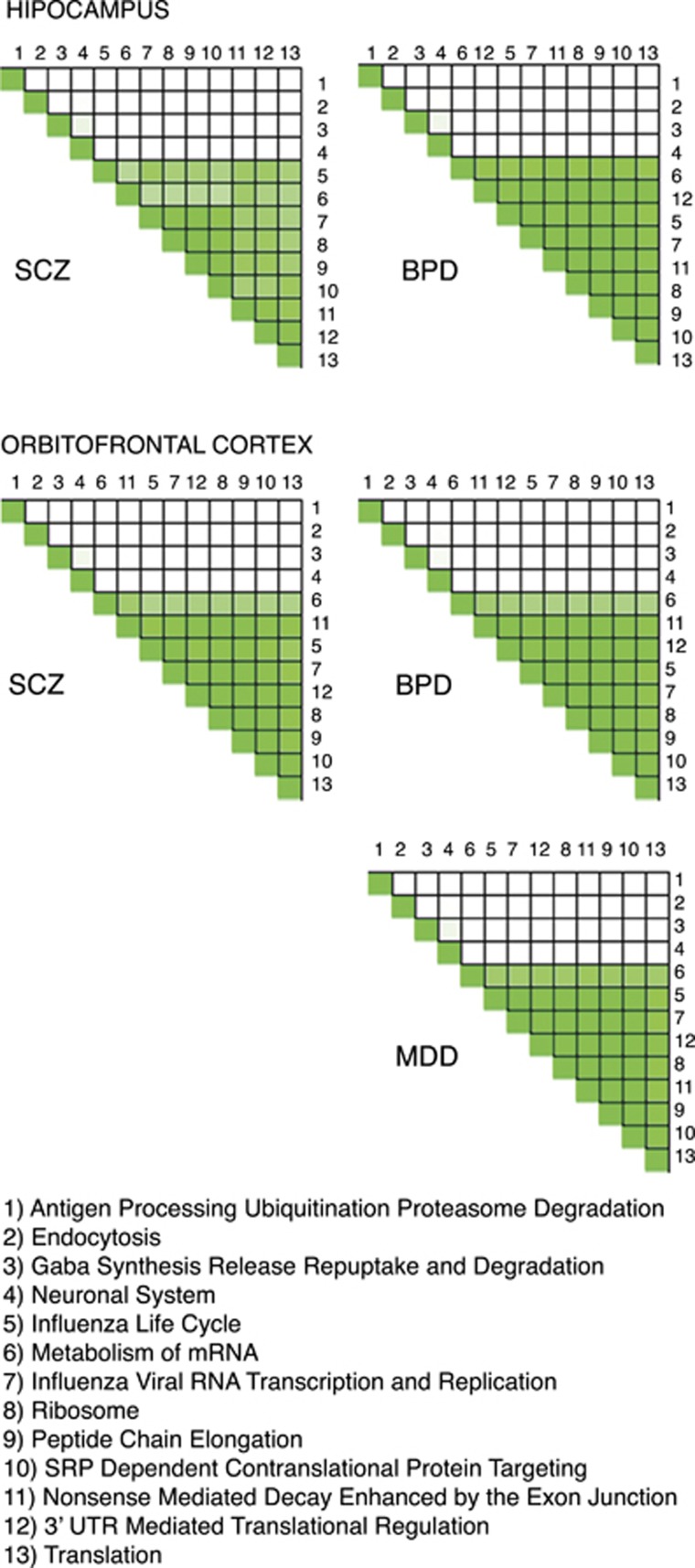
Overlap between lists of genes driving enrichment of gene sets. Darker green indicates higher degree of overlap. As different genes may contribute to enrichment of the gene sets in each sample group, separate heat map is plotted for each comparison. The top two plots denote genes with increased expression in SCZ and BPD relative to control in the hippocampus, while the bottom three plots denote genes with increased expression in SCZ, BPD and MDD in the OFC. BPD, bipolar disorder; MDD, major depressive disorder; mRNA, messenger RNA; OFC, orbitofrontal cortex; SCZ, schizophrenia; UTR, untranslated region.

To identify the genes that drive enrichment of each universal gene set, we performed a leading edge analysis in GSEA^40^ (Supplementary Tables S18–S23, Figure 2). Many of the same genes drive enrichment of all of the ribosomal gene sets. However, additional genes that are not part of the KEGG Ribosome set also drive enrichment of related sets with specialized functions. Supplementary Tables S18–S22 list the genes in each analysis that drive enrichment of the universal gene sets. Depending on the diagnostic group and brain region, between 135 and 218 genes drive expression of at least one of the ribosomal gene sets. Of these, between 49 and 70 (26–52% of the related genes in each analysis) are present in all the nine sets, whereas between 27 and 60% drive enrichment of only one set (Supplementary Table S23). In contrast, there is very little overlap between the genes driving enrichment of the four sets with decreased expression in BPD, MDD and SCZ (Figure 2). In each of the five analyses, between 333 and 366 genes drive enrichment of the four sets, with no genes driving enrichment of all four sets and 17 to 22 genes driving more than one set (Supplementary Tables S18–S23). The most strongly downregulated are neuronal system genes, followed by genes involved in antigen processing, GABA signaling and endocytosis (Table 3).

In addition to the 13 universal gene sets, 35 sets were enriched in all mood disorder samples but not SCZ (Supplementary Table S17) including genes involved in synaptic signaling, translation, opioid signaling, transfer RNA synthesis and MHC class II antigen presentation. Because of the large number of gene sets that are enriched in all mood disorder samples, we further examined the overlap between BPD and MDD in the OFC. Although only two of the 26 genes that were differentially expressed in MDD were also significant in BPD, 182 (71%) of the 257 gene sets that are enriched in MDD were enriched in the same direction in BPD. As all the OFC samples were compared with the same controls, we also examined the overlap between BPD and SCZ in the OFC. Of 216 gene sets enriched in SCZ, 83 (38%) were enriched in the same direction in BPD.

In addition to the shared gene sets, 19 additional sets replicated in BPD but were not enriched in MDD (Supplementary Table S16). These include genes implicated in Alzheimer's and Huntington's diseases, as well as phosphatidylinositol signaling, aquaporin-mediated transport, adipocytokine signaling and regulation of the cell cycle. Six gene sets replicated in SCZ alone, including olfactory signaling, class I MHC-mediated antigen processing and membrane trafficking (Supplementary Table S16).

## Discussion

We identified gene sets describing key biological processes with perturbed expression in SCZ and BPD in the postmortem hippocampus, then replicated our results in the OFC of a separate cohort. Our initial analysis included high-depth RNA-seq of 100 hippocampal samples from demographically well-matched individuals from the Stanley Array Collection and we replicated our findings in 57 OFC samples from the Stanley Neuropathology Consortium. We identified 13 gene sets that replicated across brain regions in SCZ and BPD, and were enriched MDD. The strongest enrichment scores were for nine sets describing functions related to mRNA processing, ribosome biogenesis and translation, all of which were overexpressed in SCZ, BPD and MDD. In addition, gene sets describing neuronal systems, antigen degradation, GABAergic signaling and endocytosis had decreased expression in all three of the psychiatric disorders. These gene sets describe processes previously implicated in SCZ, BPD or MDD by others using RNA-seq,^6, 8, 9, 30, 47^ microarrays^6, 10, 11, 12, 48^ or protein profiling^49, 50^ of postmortem brain samples. Our findings establish the central importance of these pathways by demonstrating that they are consistently altered in all diagnosis groups in two brain regions from two cohorts.

Our approach is aimed at identifying the core molecular deficits associated with psychiatric disease, so we added an additional level of stringency by using different brain regions from each cohort. The rationale for this approach is based on the fact that disease-associated genetic variants that regulate gene expression (that is, eQTLs) are present in every cell and therefore likely alter gene expression in multiple brain regions. Accordingly, altered gene expression in many brain regions has been reported in psychiatric disease.^7, 8, 9, 10, 11, 12, 13, 27, 28, 29, 30, 31, 32, 33, 34, 35^ Although we do not suggest that all changes in gene expression associated with disease will be consistent across brain regions, we provide evidence for the existence of robust changes in expression of certain gene pathways in multiple brain regions. Just as co-expression modules that are preserved across the brain and blood are driven by genes with more heritable gene expression levels than modules that are tissue specific,^39^ the changes in gene expression that are consistent across brain regions are more likely to represent core molecular deficits that are fundamental to disease development. Although our hypothesis is that the gene pathways we identified are likely disrupted in multiple brain regions, it is important to note that both the hippocampus and the OFC encode parallel but interactive cognitive maps.^51^ Potentially, the molecular deficits we describe might specifically disrupt such functions in the OFC and the hippocampus and thus contribute to the cognitive deficits observed in psychiatric disorders.

In addition to being present throughout the brain, many genetic variants implicated in psychiatric disease associate with multiple disorders.^1, 5^ The fact that we were able to identify gene pathways that are consistently dysregulated in different disorders as well as across brain regions and cohorts supports the hypothesis that a core set of molecular deficits may exist in psychiatric disorders. The limitation of this approach is that we may miss changes that are specific to a single brain region or disorder. Any such changes are likely included among the results presented in the supplement, but further replication studies in the hippocampus and OFC are needed to identify such differences.

Our results are consistent with findings from GWAS, which conclude that many genes of small effect size contribute to the pathology of psychiatric disorders. Similar to previous postmortem expression studies, we find subtle differences in the expression levels of individual genes between psychiatric cases and unaffected controls.^8, 29, 30^ Using gene set enrichment analysis, which measures small but consistent changes in expression of genes that work together in a biological pathway,^40^ we identified a number of gene pathways that appear to be consistently altered in psychiatric disorders. Each individual comparison, for example, SCZ vs CONTROL in hippocampus, yields many enriched gene sets but, based on our functional network analysis of the enriched gene sets, most describe related pathways and their enrichment is driven by an overlapping set of genes. The fact that expression of multiple gene pathways is affected in psychiatric disease is not surprising given that there are 108 different genetic regions associated with SCZ^4^ alone and a polygenic risk score is the most accurate predictor of psychiatric disease.^52^ In addition, disease-associated polymorphisms are enriched in genes involved in posttranslational modification of histones, a key regulator of transcription in the cell.^3^ Thus, our expression results are highly congruent with genetic findings and support the notion that many genes of small effect size contribute to the pathology of psychiatric disorders.

This study differs from previous RNA-seq studies^8, 29, 30^ that identified genes that are dysregulated in SCZ and BPD using different samples from the Stanley brain collections. We provide RNA sequencing results from brain regions that have not previously been sequenced and apply analysis methods designed to identify the overarching trends in gene expression that are in common across brain regions. Kim *et al.*^8^ and Zhao *et al.*^30^ both noted difficulty in replicating differential expression of individual genes. As both studies utilized hypergeometric tests for pathway enrichment based on significant genes, the lack of replication at the gene level proved problematic. Kim *et al.* therefore focused on gene–gene interactions revealed by co-expression analysis, whereas Zhao *et al.* selected the genes from the replication set with the greatest expression differences although none reached significance. We instead used expression information from all the genes to identify pathways enriched in each diagnostic group using GSEA. We then replicated the gene set enrichment in a different brain region from an additional cohort and identified validated pathways that are in common across multiple diagnostic groups.

We accounted for the effects of sex, age, brain pH and PMI by including these variables as covariates while determining differential expression of each gene. Although we were not able to specifically test the effect of medications on gene set enrichment, we accounted for antipsychotic use by evaluating gene expression in each diagnostic group independently and focusing on gene sets that were universally perturbed. All of the gene sets that replicated in both SCZ and BPD were also enriched in MDD although the MDD group did not use antipsychotics. Similarly, more individuals in the MDD group than in the BPD and SCZ groups took antidepressants. Further confidence that our results are not driven by medication use comes from a comparison of gene expression in postmortem brains^10^ in which subjects with BPD exposed to antipsychotics had gene expression levels that were closer to those of control individuals than medication-naive patients.

The nine gene sets overexpressed in SCZ, BPD and MDD all contain genes from the KEGG ribosome set. Each set also contains additional dysregulated genes, suggesting that multiple functional pathways involving ribosomes are affected in the three target disorders. Affected processes include ‘nonsense mediated decay enhanced by the exon junction complex', ‘metabolism of mRNA', ‘viral RNA transcription and replication', ‘3′-UTR (untranslated region)-mediated translational regulation', ‘peptide chain elongation' and ‘translation'. Our results indicate that the magnitude and significance of overexpression of ribosomal genes is greater in mood disorder samples than SCZ, but is significant in all the three disorders. Increased expression of ribosomal genes in SCZ has also been noted in other recent studies. ‘Translational elongation', ‘ribosome biogenesis' and ‘ribosomal RNA processing' gene sets were enriched in a co-expression module in the hippocampus with increased expression in SCZ,^8^ and the ‘ribosome', ‘translation', ‘translational elongation' and the 'ribosomal subunit' GO terms were enriched among genes differentially expressed in SCZ in the superior temporal gyrus.^13^ Also, protein expression profiling of human induced pluripotent stem cell-derived neural progenitor cells revealed increased levels of ribosomal proteins and translation factors as well as increased translational activity and total protein levels in cells from SCZ patients relative to controls.^53^ In addition, co-expression modules associated with BPD in the dorsolateral prefrontal cortex that included both upregulated and downregulated genes were enriched for ‘RNA processing' and ‘ribosomal subunit' GO terms,^9^ and ribosomal genes were overexpressed in the hippocampus of mice modeling a *Creb1* promoter mutation that causes familial MDD.^54^ Several forms of synaptic plasticity may require the activation of stalled polyribosomes that have initiated translation and are paused before peptide elongation.^55, 56^ Intriguingly, ketamine and other rapid-acting antidepressants work in part via phosphorylation cascades triggered by the mTOR pathway that ultimately stimulate translation of synaptogenic proteins,^57^ suggesting that translational deficits may exist in untreated individuals. It will, therefore, be interesting to study whether increased transcription of ribosomal protein genes in the hippocampus and OFC correlates with increased translation or with a buildup of stalled polyribosomes.

Many of the gene sets that we found to have decreased expression in SCZ, BPD and MDD also overlap with genes previously implicated in one or two disorders. The ‘Reactome Neuronal Systems' gene set contains ‘synaptic proteins' genes that are differentially co-regulated in BPD and MDD in the hippocampus^8^ and dysregulated in BPD in the premotor cortex.^10^ In the prefrontal cortex, genes involved in nervous system development,^12^ synaptic density and plasticity^30^ have altered expression in SCZ and BPD. In addition, genes that impact long-term potentiation have altered expression in the anterior cingulate cortex in SCZ.^49^ The role of GABAergic signaling in SCZ is also established,^6, 8, 47, 58^ consistent with our findings. Defective regulation of antigen processing and ubiquitination is supported by the finding that ubiquitin-mediated degradation genes are dysregulated in BPD^48^ and by protein expression differences in ubiquitination and degradation pathways in the hippocampus in SCZ and BPD.^50^ Dysregulation of ‘endocytosis' genes is supported by the previous finding that proteins in the endocytosis pathway have altered expression in SCZ in the postsynaptic density^49^ and gene pathways such as ‘Fc gamma receptor-mediated phagocytosis' are dysregulated in BPD and SCZ in the cingulate cortex.^30^ Together, ‘antigen processing via ubiquitination-mediated proteasome degradation' to make class I MHC molecules and ‘endocytosis' in the form of Fc gamma receptor-mediated phagocytosis contribute to the adaptive immune system, which has repeatedly been implicated in SCZ^59, 60^ and MDD.^61^ Markers of inflammation and innate immunity also frequently have altered expression in psychiatric disorders.^6, 8, 12, 59, 60, 61^ We found that gene sets related to innate immunological processes such as cytokine signaling were enriched in SCZ, BPD and MDD in both the OFC and the hippocampus, but the direction of the differential expression was not consistent between brain regions or diagnostic groups. Accordingly, while dysregulation of innate immunity has been established in both SCZ and BPD, expression of innate immunity genes represents a major difference between the diseases.^8, 11^

Our observation that key pathways are concordantly regulated in SCZ, BPD and MDD is consistent with GWAS results as polygenic risk scores for SCZ, BPD and MDD show some cross-disorder association^1^ and disease-associated loci suggest the involvement of immune and neuronal signaling in all the three disorders.^3^ In particular, the MHC locus, which encodes components of the MHC Class I and II complexes, is strongly associated with SCZ and BPD^62^ and key genes within the locus have been shown to have decreased expression in SCZ and BPD throughout the brain.^63^ Accordingly, the Reactome MHC Class II antigen presentation gene set had decreased expression in all mood disorder samples, whereas the Reactome Class I MHC-mediated antigen processing presentation gene set had decreased expression in SCZ. Calcium signaling has also been implicated in SCZ, BPD and MDD based on GWAS. We found that dysregulation of calcium signaling genes was reproducible in both SCZ and BPD, but expression was increased in SCZ and decreased in mood disorder samples. As genetic variation can affect gene expression in a variety of ways, our findings fit with the GWAS results and may indicate that some variants have a context-specific impact on gene expression.

Although no single gene was differentially expressed in the same disorder in both brain regions, *MT1X*, *SLC11A1, S100A8* and *NR4A2* were significant in SCZ in the hippocampus and nominally significant in the OFC. In addition, *MT1X* and *FKBP5* reached nominal significance in BPD in the hippocampus and were significant in the OFC. *MT1X* encodes a metallothionein that may be neuroprotective during stress response and is dysregulated in postmortem brains in psychosis^64^ and suicide.^65^ *SLC11A1* encodes an iron transporter with a key role in innate immunity.^66^ *S100A8* encodes a calcium-binding protein that regulates inflammation and immune response and has altered expression in blood from SCZ patients.^67^ *NR4A2* encodes a transcription factor regulating dopaminergic neurosynthesis^68^ previously shown by *in situ* hybridization and western blotting to have decreased expression in postmortem brains in psychiatric disease.^69, 70^ *FKBP5* encodes a key regulator of glucocorticoid response, is widely implicated in psychiatric disease and was previously identified as overexpressed in the frontal cortex in SCZ, BPD and MDD.^71, 72^

We performed one of the largest RNA sequencing studies of postmortem brain tissue in psychiatric disease and discovered that specific gene sets are dysregulated across cohorts, brain regions and disorders. The fact that the gene sets identified in this study are consistently altered across brain regions suggests that they may represent fundamental molecular deficits associated with psychiatric disease. The further characterization of biological processes described by these gene sets has the potential to reveal important insights into disease pathology and may finally unravel the etiology of psychiatric disorders.

## Figures and Tables

**Table 1:** Demographic information for samples included in gene expression analysis

*Group*	*Hippocampus (cohort 1)*			*Orbitofrontal cortex (replication cohort)*			
	*SCZ*	*BPD*	*Controls*	*SCZ*	*BPD*	*MDD*	*Controls*
Number	35	33	32	13	14	15	15
Age	42.6	45.4C	43.9	44.65	41.9	46.5	48.1
Age range	19–59	19–64	31–60	25–62	25–61	30–65	29–68
Sex	9F, 26M	18F, 15M	9F, 24M	8M, 5F	9M, 5F	9M, 6F	9M, 6F
Race	34C, 1H	32C, 1NA	32C	10C, 3A	13C, 1AA	15C	14C, 1AA
PMI (h)	31.4	38	30	35.8	30.9	27.5	23.7
PMI range	9–80	12–84	9–58	12–61	13–62	7–47	8–42
pH	6.5	6.4	6.6	6.2	6.2	6.2	6.3
pH range	5.9–6.9	5.8–7	6–7	5.8–6.6	5.8–6.5	5.8–6.5	5.8–6.6
Side	17L, 18R	18L, 15R	15L, 17R	8L, 5R	6L, 8R	9L, 6R	8L, 7R
Mood stabilizers	10Y, 24N	21Y, 11N, 1NA	32N	3Y, 10N	9Y, 5N	2Y, 13N	15N
Antidepressants	9Y, 25N	19Y, 13N, 1NA	32N	5Y, 8N	7Y, 7N	127, 3N	15N
Antipsychotics	35Y	22Y, 11N	32N	12Y, 1N	11Y, 3N	15N	15N
Lifetime dosage	85 004	9915	0	61 077	20 032	0	0
Dosage range	50–400 000	0–130 000	0	0–150 000	0–60 000	0	0

Abbreviations: BPD, bipolar disorder; F, female; L, left; M, male; MDD, major depressive disorder; NA, not available; PMI, postmortem interval; R, right; SCZ, schizophrenia.

Information on matched traits for hippocampus samples (*N*=100 after removing outliers) from the Stanley Microarray Collection and OFC samples (*N*=57 after removing outliers) from the Stanley Neuropathology Consortium Collection that were included in the differential expression analysis. 'AA' represents African American, 'A' represents Asian, 'C' represents Caucasian, 'H' represents Hispanic. ‘Mood Stabilizers', ‘Antidepressants' and ‘Antipsychotics' are listed as 'Y' if there is a record of an individual having ever taken one and 'N' if there is no such record. ‘Lifetime dosage' is the mean estimated lifetime dosage of antipsychotics in fluphenazine equivalents (mg). ‘Dosage range' is the lowest and highest lifetime dosages of antipsychotics of all individuals in each group.

**Table 2:** Genes differentially expressed in the hippocampus in schizophrenia and bipolar disorder

*Gene*	*All*	*Schizophrenia vs control*					*Bipolar disorder vs control*				
	*Counts*	*log*_*2*_*FC*	*lfcSE*	*Wald*	P-*value*	*MTC* P	*log*_*2*_*FC*	*lfcSE*	*Wald*	P-*value*	*MTC* P
*LOC100131257*	219.38	0.41	0.08	5.43	5.52E−08	**9.06E−04**	0.1	0.07	1.38	1.69E−01	1
*LOC100288637*	309.86	−0.38	0.08	−4.94	7.80E−07	**4.27E−03**	−0.31	0.08	−4.06	4.92E−05	0.12
*KIF19*	1732.95	0.46	0.09	5.01	5.50E−07	**4.27E−03**	0.18	0.09	1.97	4.88E−02	1
*SLC11A1*	304.43	0.42	0.09	4.77	1.82E−06	**7.48E−03**	0	0.09	0.04	9.67E−01	1
*FKBP5*	2769.46	0.41	0.09	4.34	1.40E−05	**3.29E−02**	0.29	0.09	3.12	1.78E−03	0.88
*GNA14*	599.69	0.38	0.09	4.35	1.36E−05	**3.29E−02**	0.07	0.09	0.78	4.35E−01	1
*LOC100188947*	150.82	0.35	0.08	4.41	1.01E−05	**3.29E−02**	0.11	0.08	1.38	1.68E−01	1
*HSPA7*	867.94	−0.28	0.07	−4.21	2.54E−05	**3.94E−02**	−0.26	0.06	−4.09	4.38E−05	0.12
*SVEP1*	715.2	0.38	0.09	4.24	2.27E−05	**3.94E−02**	0.29	0.09	3.27	1.08E−03	0.71
*MT1X*	5020.4	0.37	0.09	4.2	2.64E−05	**3.94E−02**	0.21	0.09	2.36	1.83E−02	1
*S100A8*	92.33	0.21	0.05	4.23	2.36E−05	**3.94E−02**	0.03	0.05	0.64	5.22E−01	1
*NR4A2*	1860.74	−0.37	0.09	−3.97	7.16E−05	**9.80E−02**	−0.29	0.09	−3.1	1.92E−03	0.89
*RPPH1*	1246.73	−0.01	0.07	−0.18	8.60E−01	1	0.48	0.06	7.39	1.51E−13	**3.29E−09**
*SCARNA2*	192.55	−0.01	0.06	−0.11	9.15E−01	1	0.35	0.06	6.12	9.48E−10	**1.04E−05**
*CDR1*	2177.67	0.02	0.08	0.23	8.20E−01	1	0.48	0.08	5.88	4.08E−09	**2.97E−05**
*PAR5*	333.34	0.08	0.09	0.89	3.73E−01	1	0.47	0.09	5.42	5.82E−08	**3.18E−04**
*RMRP*	604.09	−0.01	0.05	−0.28	7.81E−01	1	0.23	0.05	5.12	2.98E−07	**1.30E−03**
*MTRNR2L6*	9.75	0.06	0.07	0.93	3.52E−01	NA	0.3	0.06	4.72	2.37E−06	**8.63E−03**
*MUC16*	10.41	0	0.04	−0.1	9.24E−01	NA	0.17	0.04	4.18	2.96E−05	**9.24E−02**

Abbreviation: NA, not available.

Counts is the average number of normalized read counts per sample; log_2_FC is log_2_ transformed fold change; lfcSE is the standard error of the log_2_ transformed fold change; Wald is the test statistic for differential expression; *P*-value is the nominal *P*-value of differential expression; MTC *P* is the adjusted *P*-value after multiple testing correction. Bold values are those that are significant after multiple testing correction (MTC *P*).

**Table 3:** Gene sets that replicated in both SCZ and BPD and were also enriched in MDD

*Gene set*	*Size*	*SCZ*				*BPD*				*MDD*	
		*Hippocampus (cohort 1)*		*Orbitofrontal (replication)*		*Hippocampus (cohort 1)*		*Orbitofrontal (replication)*		*Orbitofrontal*	
		*NES*	*FDR*	*NES*	*FDR*	*NES*	*FDR*	*NES*	*FDR*	*NES*	*FDR*
Reactome peptide chain elongation	85	2.79	0.002	2.88	0	7.06	0	7.17	0	8.21	0
KEGG ribosome	86	2.7	0.003	3.07	0	6.88	0	7.31	0	7.93	0
Reactome influenza viral RNA transcription and replication	101	2.44	0.007	2.77	0.001	6.55	0	6.9	0	7.9	0
Reactome nonsense mediated decay enhanced by the exon junction complex	106	2.66	0.003	2.66	0.001	6.1	0	6.38	0	7.35	0
Reactome 3′-UTR mediated translational regulation	104	2.69	0.003	2.53	0.003	6.37	0	6.49	0	7.31	0
Reactome SRP-dependent cotranslational protein targeting to membrane	108	2.43	0.008	2.36	0.008	5.69	0	6.7	0	7.16	0
Reactome translation	144	2.38	0.009	2.18	0.017	5.25	0	6.1	0	6.62	0
Reactome influenza life cycle	135	2.6	0.003	2.06	0.031	5.67	0	5.72	0	6.34	0
Reactome metabolism of mRNA	208	2.65	0.003	2.41	0.006	4.21	0	5.47	0	5.72	0
KEGG endocytosis	179	−3.6	0	−2.42	0.005	−2.56	0.002	−2.46	0.001	−2.2	0.01
Reactome antigen processing ubiquitination proteasome degradation	199	−2.47	0.005	−2.74	0.001	−3.47	0	−2.28	0.004	−2.94	0
Reactome GABA synthesis release reuptake and degradation	17	−2.7	0.001	−1.88	0.047	−2.75	0.001	−2.36	0.002	−3.04	0
Reactome neuronal system	276	−2.26	0.021	−2.71	0.001	−3.9	0	−7.17	0	−6.17	0

Abbreviations: BPD, bipolar disorder; FDR, false discovery rate *q*-value for enrichment of each gene set in each of the five sample groups; GSEA, gene set enrichment analysis; KEGG, Kyoto Encyclopedia of Genes and Genomes; mRNA, messenger RNA; MDD, major depressive disorder; NES, GSEA set-size normalized enrichment scores for each set; SCZ, schizophrenia; SRP, signal recognition particle; UTR, untranslated region.
