# Supplementary material for: Consistently altered expression of gene sets in postmortem brains of individuals with major psychiatric disorders
Source: Transl Psychiatry. 2016 Sep 13;6(9):e890–. doi: 10.1038/tp.2016.173 (PMC5048210; doi:10.1038/tp.2016.173)
Supplement: Supplementary Table Legends [file tp2016173x1.docx]

**Supplementary Table Legends**

**Table S1) RNA sequencing read totals for hippocampal samples**. Qualty metrics from TopHat2 alignment to the human genome (hg19). The columns denote B) The number of first strand reads in each fastq file; C) The number of second strand reads in each fastq file; D) The total number of reads from both fastq files that aligned to the genome; E) The number of first strand reads that aligned; F) The number of second strand reads that aligned; G) The number of first and second strand reads that met all of the restraits for proper pairing, i.e. orientation, distance, and chromosome; H) The number of reads where both reads in the pair mapped; I) The number of pairs where only one of the reads mapped, J) The number of pairs where the reads mapped to different chromosomes, K) The number of pairs that mapped to different chromosomes with a mapping quality score greater than 5

**Table S2) RNA sequencing read totals for orbitofrontal cortex samples**. Qualty metrics from TopHat2 alignment to the human genome (hg19). The columns denote B) The number of first strand reads in each fastq file, C) The number of reads that aligned to the genome

**Table S3)** **Gene sets enriched in SCZ in the hippocampus at an FDR < 0.05**. Size refers to the number of genes in the expression dataset. ES is the enrichment score. NES is the normalized enrichment score. FDR is the FDR q value for enrichment of each gene set.

**Table S4) Gene sets enriched in BPD in the hippocampus at an FDR < 0.05**. Size refers to the number of genes in the expression dataset. ES is the enrichment score. NES is the normalized enrichment score. FDR is the FDR q value for enrichment of each gene set.

**Table S5) Table of overlap between gene sets signifcantly enriched in SCZ in the hippocampus.** Interaction name refers to the two gene sets that are being compared. The overlap coefficient between the two gene sets is equal to the number of shared genes divided by the total size of the smaller of the two gene sets. Overlap size is equal to the number of shared genes. Overlap genes are the genes that are in common between the two gene sets.

**Table S6) Table of overlap between gene sets signifcantly enriched in BPD in the hippocampus.** Interaction name refers to the two gene sets that are being compared. The overlap coefficient between the two gene sets is equal to the number of shared genes divided by the total size of the smaller of the two gene sets. Overlap size is equal to the number of shared genes. Overlap genes are the genes that are in common between the two gene sets.

**Table S7) Genes differentially expressed in BPD in the orbitofrontal cortex:** Differential expression results from DESeq2 for all significant genes. Base mean denotes normalized number of reads per sample aligning to each gene. Log2 FC is the log2 transformed fold change in expression in bipolar disorder relative to control. Lfc SE is the standard error of the log transformed fold change. Wald is the wald test statistic for differential expression of each gene. P value is the nominal p value for differential expression. MTC p is the multiple testing corrected p value for differential expression.

**Table S8) Genes differentially expressed in MDD in the orbitofrontal cortex**: Differential expression results from DESeq2 for all significant genes. Base mean denotes normalized number of reads per sample aligning to each gene. Log2 FC is the log2 transformed fold change in expression in major depression relative to control. Lfc SE is the standard error of the log transformed fold change. Wald is the wald test statistic for differential expression of each gene. P value is the nominal p value for differential expression. MTC p is the multiple testing corrected p value for differential expression.

**Table S9) Expression in the hippocampus and orbitofrontal cortex of all genes that are differentially expressed in the hippocampus**. Expression in each diagnostic group relative to control is shown for both the hippocampus and orbitofrontal cortex. For each tissue type, base mean denotes normalized number of reads per sample aligning to each gene. Log2 FC is the log2 transformed fold change in expression in bipolar disorder relative to control. Lfc SE is the standard error of the log transformed fold change. Wald is the wald test statistic for differential expression of each gene. P value is the nominal p value for differential expression. MTC p is the multiple testing corrected p value for differential expression.

**Table S10) Gene sets enriched in SCZ in the orbitofrontal cortex at an FDR < 0.05**. Size refers to the number of genes in the expression dataset. ES is the enrichment score. NES is the normalized enrichment score. FDR is the FDR q value for enrichment of each gene set.

**Table S11) Gene sets enriched in BPD in the orbitofrontal cortex at an FDR < 0.05**. Size refers to the number of genes in the expression dataset. ES is the enrichment score. NES is the normalized enrichment score. FDR is the FDR q value for enrichment of each gene set.

**Table S12) Gene sets enriched in MDD in the orbitofrontal cortex at an FDR < 0.05**. Size refers to the number of genes in the expression dataset. ES is the enrichment score. NES is the normalized enrichment score. FDR is the FDR q value for enrichment of each gene set.

**Table S13) Table of overlap between gene sets signifcantly enriched in SCZ in the orbitofrontal cortex.** Interaction name refers to the two gene sets that are being compared. The overlap coefficient between the two gene sets is equal to the number of shared genes divided by the total size of the smaller of the two gene sets. Overlap size is equal to the number of shared genes. Overlap genes are the genes that are in common between the two gene sets.

**Table S14) Table of overlap between gene sets signifcantly enriched in BPD in the orbitofrontal cortex.** Interaction name refers to the two gene sets that are being compared. The overlap coefficient between the two gene sets is equal to the number of shared genes divided by the total size of the smaller of the two gene sets. Overlap size is equal to the number of shared genes. Overlap genes are the genes that are in common between the two gene sets.

**Table S15) Table of overlap between gene sets signifcantly enriched in MDD in the orbitofrontal cortex.** Interaction name refers to the two gene sets that are being compared. The overlap coefficient between the two gene sets is equal to the number of shared genes divided by the total size of the smaller of the two gene sets. Overlap size is equal to the number of shared genes. Overlap genes are the genes that are in common between the two gene sets.

**Table S16) Gene sets that were replicated in BPD or SCZ alone**. Gene sets enriched in BPD were not also enriched in MDD. Size refers to the number of genes in the expression dataset. ES is the enrichment score. NES is the normalized enrichment score. FDR is the FDR q value for enrichment of each gene set.

**Table S17) Gene sets that were replicated in BPD and also enriched in MDD**. Size refers to the number of genes in the expression dataset. ES is the enrichment score. NES is the normalized enrichment score. FDR is the FDR q value for enrichment of each gene set.

**Table S18) Leading edge genes that contribute to enrichment of the 13 universal gene sets in SCZ in the hippocampus**. Each column heading indicates a gene set with the leading edge genes listed below.

**Table S19) Leading edge genes that contribute to enrichment of the 13 universal gene sets in BPD in the hippocampus**. Each column heading indicates a gene set with the leading edge genes listed below.

**Table S20) Leading edge genes that contribute to enrichment of the 13 universal gene sets in SCZ in the orbitofrontal cortex**. Each column heading indicates a gene set with the leading edge genes listed below.

**Table S21) Leading edge genes that contribute to enrichment of the 13 universal gene sets in BPD in the orbitofrontal cortex**. Each column heading indicates a gene set with the leading edge genes listed below.

**Table S22) Leading edge genes that contribute to enrichment of the 13 universal gene sets in MDD in the orbitofrontal cortex**. Each column heading indicates a gene set with the leading edge genes listed below.

**Table S23) Number of Leading Edge Genes Driving Enrichment of Universal Gene Sets.** Total = the number of genes driving all 13 gene sets; Ribosomal = the number of genes diving enrichment of the 9 gene sets related to ribosomal functions; In 9 Ribosomal = Number of genes in leading edge of all nine related gene sets; >1 Ribosomal = number of genes driving expression of more than one of the 9 gene sets; Downregulated = number of genes driving enrichment of the four universally downregulated gene sets; In all Downregulated = number of leading edge genes in all four sets; >1 Downregulated = number of leading edge genes driving enrichment of more than one of the downregulated gene sets.
