# Supplementary material for: Consistently altered expression of gene sets in postmortem brains of individuals with major psychiatric disorders
Source: Transl Psychiatry. 2016 Sep 13;6(9):e890–. doi: 10.1038/tp.2016.173 (PMC5048210; doi:10.1038/tp.2016.173)
Supplement: Supplementary Figure Legends [file tp2016173x2.docx]

**Supplementary Figure Legends**

**Figure S1) Principal components analyses showing sample-to-sample distances based on global differences in gene expression**. Outliers 1 and 2 denoted by arrows are the two samples in each set that contribute most to the total variation between samples. A and C show all samples while B and D show the overall variation gene expression after the first outlier was removed from each analysis.

**Figure S2) Distribution of differential expression p values**. Histograms showing the number of genes in each analysis at each p value range after correction for multiple testing. Values between 0 and 1 are represented by 20 columns, each representing the number of genes with a value of p to p + 0.05 except when SCZ was compared to control in the OFC. In OFC all genes had a corrected p value of 1 for differential expression in SCZ.

**Figure S3) Functional network analysis of overlap between gene sets enriched in SCZ in the hippocampus.** Red circles denote gene sets with increased expression affected individuals while blue circles indicate decreased expression. The size of each circle represents the number of genes from our gene expression datasets that are annotated in each gene set. The thickness of the lines connecting two circles represents the proportion of genes that are in common between the two gene sets.

**Figure S4) Functional network analysis of overlap between gene sets enriched in BPD in the hippocampus.** Red circles denote gene sets with increased expression affected individuals while blue circles indicate decreased expression. The size of each circle represents the number of genes from our gene expression datasets that are annotated in each gene set. The thickness of the lines connecting two circles represents the proportion of genes that are in common between the two gene sets.

**Figure S5) Functional network analysis of overlap between gene sets enriched in SCZ in the orbitofrontal cortex.** Red circles denote gene sets with increased expression affected individuals while blue circles indicate decreased expression. The size of each circle represents the number of genes from our gene expression datasets that are annotated in each gene set. The thickness of the lines connecting two circles represents the proportion of genes that are in common between the two gene sets.

**Figure S6) Functional network analysis of overlap between gene sets enriched in BPD in the orbitofrontal cortex.** Red circles denote gene sets with increased expression affected individuals while blue circles indicate decreased expression. The size of each circle represents the number of genes from our gene expression datasets that are annotated in each gene set. The thickness of the lines connecting two circles represents the proportion of genes that are in common between the two gene sets.

**Figure S7) Functional network analysis of overlap between gene sets enriched in MDD in the orbitofrontal cortex.** Red circles denote gene sets with increased expression affected individuals while blue circles indicate decreased expression. The size of each circle represents the number of genes from our expression datasets that are annotated in each gene set. The thickness of the lines connecting two circles represents the proportion of genes that are in common between the two gene sets.

**Figure S8) Gene Sets Enriched in SCZ, BPD, and MDD in the Hippocampus and Orbitofrontal Cortex.** Red circles denote gene sets with increased expression affected individuals while blue circles indicate decreased expression. The size of each circle represents the number of genes in each set while the thickness of the lines connecting two circles represents the proportion of genes that are in common between the two gene sets.
