# Supplementary material for: Consistently altered expression of gene sets in postmortem brains of individuals with major psychiatric disorders
Source: Transl Psychiatry. 2016 Sep 13;6(9):e890–. doi: 10.1038/tp.2016.173 (PMC5048210; doi:10.1038/tp.2016.173)
Supplement: Supplementary Methods [file tp2016173x3.docx]

**SUPPLEMENTAL METHODS**

**Library preparation and sequencing:**

Strand specific paired-end sequencing libraries for the hippocampus samples were prepared from 1µg of total RNA following the manufacturers recommended protocol for the Illumina TruSeq Stranded mRNA Sample Preparation Kit.

Strand specific single-end RNA-seq libraries for the orbitofrontal cortex were constructed using the TruSeq RNA SamplePrep Guide version 15008136_A with modifications. Briefly, mRNA was purified from 2 µg of total orbitofrontal cortex RNA using Illumina RNA purification beads, the resulting mRNA was fragmented using the Illumina Elute, Prime, Fragment Mix and first strand cDNA was synthesized following the TruSeq RNA protocol. Second strand cDNA was synthesized using 8 μl of 10X NEBNext® Second Strand Synthesis (dNTP-Free) Reaction Buffer, 2μl of 10X SuperScript II RT Buffer (NEB), 250µM of each dATP, dUTP, dCTP and dGTP, all of the material from the first strand cDNA reaction and 4μl of second strand enzyme (NEB) in a total of 100μl. The reaction was incubated at 16^0^ C for 2.5 hours. The resulting double stranded cDNA was purified, end repaired and adenylated following the TruSeq RNA Sample Prep protocol. One microliter of Illumina adapters were used for the ligation following the TruSeq RNA Sample Prep protocol. The adapter ligated cDNA library was then purified using Ampure beads and subjected to USER enzyme digestion in 5 μl of 10X HotStar PCR buffer (Qiagen), 1 unit of USER enzyme (NEB) in a total of 50μl. This reaction was incubated at 37^0^ C for 15 minutes and the enzyme was inactivated by heating to 95^0^ C for 5 minutes. The digested cDNA was purified using Ampure beads and PCR amplified following the protocol in the TruSeq RNA Sample Prep protocol.

**Alignment:** Reads from each sample were aligned to the human genome (hg19) using the topHat2 short read aligner (1) version 2.0.11. Paired-end libraries from the hippocampus were aligned using the options: “-r 300 --mate-std-dev 100 --library-type fr-firststrand --b2-very-sensitive”. Single-end libraries from the orbitofrontal cortex were aligned were aligned using the Tophat2 version 2.0.8 with its default settings (1).

**Outlier removal:** To assess the impact of potential batch effects and confounding variables, we performed principal components analysis based on sample-to-sample differences in global gene expression comparing all samples from each brain region to each other irrespective of diagnosis. In other words, all 102 hippocampal samples were considered as a single group (Figure S1A) and all 59 orbitofrontal cortex samples were considered as a separate group (Figure S1C). In each group one sample accounted for most of the overall variance along one of the principal components (Labeled 1 in Figure S1A and C). After the first outlier was removed from each group, the principal components of variance were again plotted (Figure S1B and D) and again one sample from each group accounted for most of the overall variance along one of the principal components (labeled 2 in Figure S1B and D). These two samples were also removed from the analysis. Following removal of the two outliers from each set, the samples remained well matched for demographic factors such as age, sex, race, postmortem interval, pH, and the side of the brain used to extract RNA (Table 1). Further information about the Stanley Medical Research Institute sample collections is available at http://www.stanleyresearch.org/brain-research/

**Gene counting** **and Differential Expression:** We counted the number of strand-specific overlaps between sequenced reads and any exon within the 23459 genes annotated in the UCSC Genome Bioinformatics hg19 knownGene.txt table using the “ShortRead” and “TxDb.Hsapiens.UCSC.hg19.knownGene” packages available at bioconductor.org. The number of reads per gene were tallied using the ShortRead summarizeOverlaps() command with the options “mode = "IntersectionStrict", ignore.strand = FALSE, inter.feature = TRUE”. For the paired-end hippocampal libraries we used the additional options “fragments = TRUE, singleEnd = FALSE”.

Following outlier removal described above, samples from all diagnosis groups in each brain region were normalized for sequencing depth and differential expression was performed using DESeq2 (2) as follows: First we created a SummarizedExperiment dataset containing all of the demographic and count information for the 100 hippocampus samples included in the analysis. We then created a DESeq dataset from the SummarizedExperiment using the command DESeqDataSet(hipSE, design= ~Sex+Age+BrainPH+PMI+Group), with hipSE referring to the dataset and “Group” referring to diagnostic group. We then used the command DESeq() to determine differences in expression associated with each disorder by comparing each diagnosis group to controls using a multi-factor design formula that included age, sex, brain pH, and postmortem interval as covariates and diagnosis as the contrast variable. Results for each comparison were accessed using the command results(desHip, contrast=c(“Group”, “SCZ”, “CTL”)) for SCZ and results(desHip, contrast=c(“Group”, “BPD”, “CTL”)) for BPD. Genes with multiple testing adjusted p values below the recommended threshold of 0.01 are reported (3). Analysis of differential expression in the orbitofrontal cortex was performed in the same manner except 57 samples were included in the analysis and an additional contrast was performed to compare MDD to CTL.

**Parameters used for Gene Set Enrichment Analysis and Functional Network Mapping:** For each diagnostic group we ranked all genes according to the Wald test statistic for differential expression provided by DESeq2^43^. Using the GseaPreranked tool in GSEA^40^ we identified gene sets that were enriched among overexpressed or under-expressed genes with a false discovery rate (FDR) q < 0.05. Using 1000 permutations, the “classic” (unweighted) enrichment statistic, and the “meandiv” normalization mode, we tested 1070 curated gene sets in the Molecular Signatures Database (c2.cp.v5.0.symbols.gmt, including KEGG, Biocarta, and Reactome canonical pathway sets) that had between 15 to 500 genes in the target lists. We utilized the EnrichmentMap^44^ application for Cytoscape^45, 46^ to map the enriched gene sets into functional networks. Gene set annotations and expression information were imported directly from the GSEA output files and mapped using the following options: Analysis type = GSEA, P-value Cutoff = 0.05, FDR Q-value Cutoff = 0.05, Similarity Cutoff= Overlap Coefficient of 0.5.

**Gene Set Enrichment Leading Edge Analysis:** Plots of the overlap between the lists of genes driving enrichment of each gene set (Figure 2) were generated using the GSEA (4) Leading Edge Analysis tool. Results were loaded into the tool, the relevant gene sets were selected, and the overlap plot was generated automatically.

1. Kim D, Pertea G, Trapnell C, Pimentel H, Kelley R, Salzberg SL. TopHat2: accurate alignment of transcriptomes in the presence of insertions, deletions and gene fusions. Genome biology. 2013;14:R36.

2. Love MI, Huber W, Anders S. Moderated estimation of fold change and dispersion for RNA-seq data with DESeq2. Genome biology. 2014;15:550.

3. Anders S, McCarthy DJ, Chen Y, Okoniewski M, Smyth GK, Huber W, Robinson MD. Count-based differential expression analysis of RNA sequencing data using R and Bioconductor. Nature protocols. 2013;8:1765-1786.

4. Subramanian A, Tamayo P, Mootha VK, Mukherjee S, Ebert BL, Gillette MA, Paulovich A, Pomeroy SL, Golub TR, Lander ES, Mesirov JP. Gene set enrichment analysis: a knowledge-based approach for interpreting genome-wide expression profiles. Proceedings of the National Academy of Sciences of the United States of America. 2005;102:15545-15550.
