# Supplementary figures and images for: Consistently altered expression of gene sets in postmortem brains of individuals with major psychiatric disorders
Source: Transl Psychiatry. 2016 Sep 13;6(9):e890–. doi: 10.1038/tp.2016.173 (PMC5048210; doi:10.1038/tp.2016.173)

Figure S2) Distribution of Differential Expression p Values

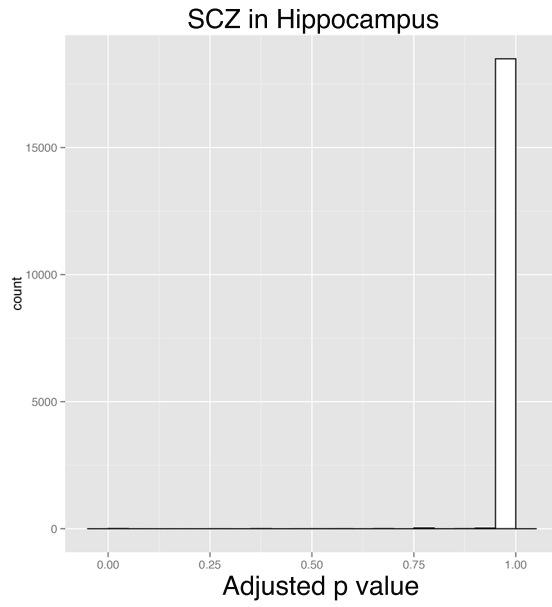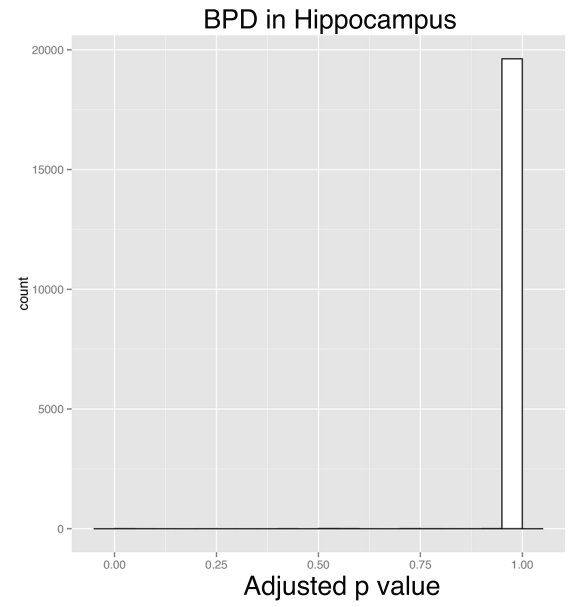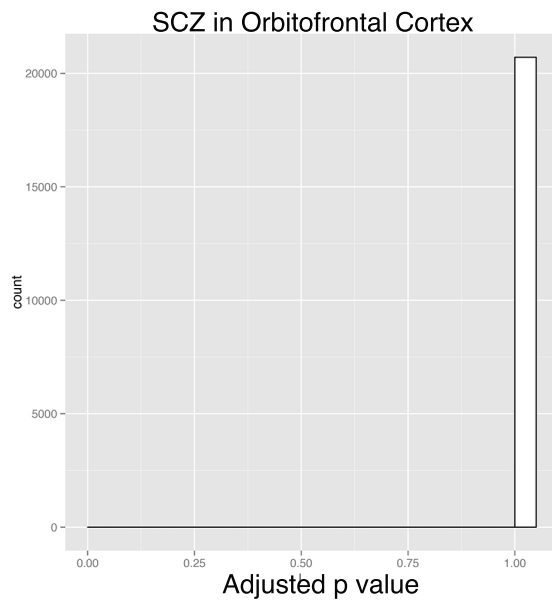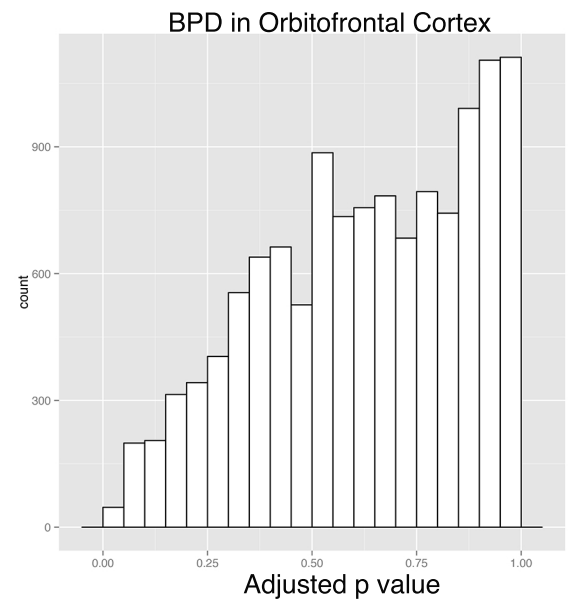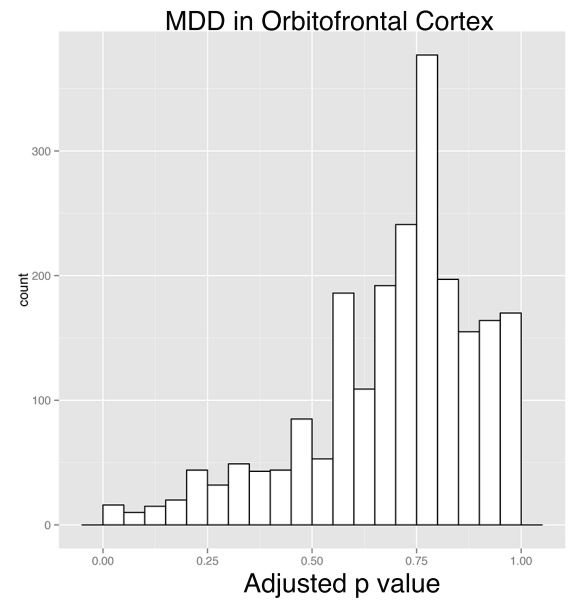

Supplement: Supplementary Figure S2 [file tp2016173x5.pdf]
