# Supplementary material for: Consistently altered expression of gene sets in postmortem brains of individuals with major psychiatric disorders
Source: Transl Psychiatry. 2016 Sep 13;6(9):e890–. doi: 10.1038/tp.2016.173 (PMC5048210; doi:10.1038/tp.2016.173)
Supplement: Supplementary Figure S6 [file tp2016173x9.pdf]

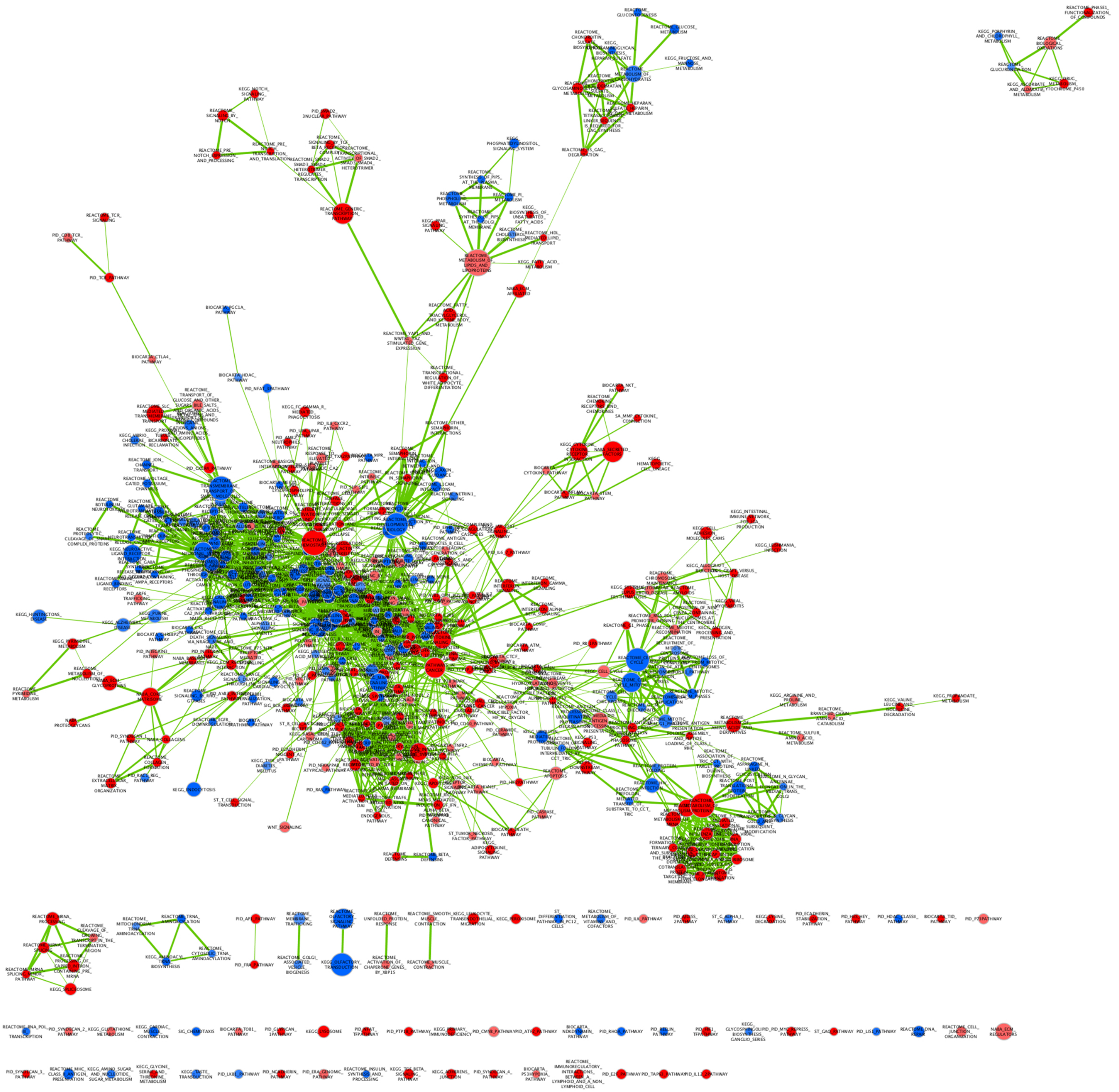

Figure S6) Functional network analysis of overlap between gene sets enriched in BPD in the orbitofrontal cortex
